# Associations between maternal pre-pregnancy BMI and infant striatal mean diffusivity

**DOI:** 10.1186/s12916-024-03340-z

**Published:** 2024-03-25

**Authors:** Aylin Rosberg, Harri Merisaari, John D. Lewis, Niloofar Hashempour, Minna Lukkarinen, Jerod M. Rasmussen, Noora M. Scheinin, Linnea Karlsson, Hasse Karlsson, Jetro J. Tuulari

**Affiliations:** 1 FinnBrain Birth Cohort Study, Turku Brain and Mind Centre, Department of Clinical Medicine, University of Turku, Turku, Finland; 2 Department of Psychiatry, Turku University Hospital and University of Turku, Turku, Finland; 3 Department of Pediatrics and Adolescent Medicine, Turku University Hospital and University of Turku, Turku, Finland; 4 Turku Collegium for Science, Medicine and Technology, University of Turku, Turku, Finland; 5 Department of Diagnostic Radiology, Turku University Hospital, Turku, Finland; 6 The Hospital for Sick Children (SickKids) Research Institute, Toronto, ON Canada; 7 Department of Pediatrics, University of California, Irvine, CA USA; 8 Centre for Population Health Research, Turku University Hospital and University of Turku, Turku, Finland; 9 Department of Psychiatry, University of Turku and Satakunta Wellbeing Services County, Turku, Finland

**Keywords:** Caudate nucleus, DTI, Maternal BMI, Mean diffusivity, Obesity, Striatum

## Abstract

**Background:**

It is well-established that parental obesity is a strong risk factor for offspring obesity. Further, a converging body of evidence now suggests that maternal weight profiles may affect the developing offspring’s brain in a manner that confers future obesity risk. Here, we investigated how pre-pregnancy maternal weight status influences the reward-related striatal areas of the offspring’s brain during in utero development.

**Methods:**

We used diffusion tensor imaging to quantify the microstructure of the striatal brain regions of interest in neonates (*N* = 116 [66 males, 50 females], mean gestational weeks at birth [39.88], SD = 1.14; at scan [43.56], SD = 1.05). Linear regression was used to test the associations between maternal pre-pregnancy body mass index (BMI) and infant striatal mean diffusivity.

**Results:**

High maternal pre-pregnancy BMI was associated with higher mean MD values in the infant’s left caudate nucleus. Results remained unchanged after the adjustment for covariates.

**Conclusions:**

In utero exposure to maternal adiposity might have a growth-impairing impact on the mean diffusivity of the infant’s left caudate nucleus. Considering the involvement of the caudate nucleus in regulating eating behavior and food-related reward processing later in life, this finding calls for further investigations to define the prognostic relevance of early-life caudate nucleus development and weight trajectories of the offspring.

**Supplementary Information:**

The online version contains supplementary material available at 10.1186/s12916-024-03340-z.

## Background

Parental obesity plays a critical role in the mental and physical health of the offspring in many stages of their lives. The existing body of research indicates that if at least one parent is obese, the children are at high risk of obesity as early as age 2 [1]. The risk is higher if the mother is obese compared to the father being obese [2].

One of the most accepted explanations for the influence of high maternal adiposity on the offspring weight status has been genetic heritability [3]. The implicated genes are mainly involved in the neural networks controlling eating habits such as caloric intake, appetite regulation, and even compulsive hyperphagia [4, 5]. However, the predictive power of genetics fluctuates over the lifespan of the offspring [6], and it does not account for the greater relation between obesity in the offspring and maternal obesity compared to paternal obesity. In addition, environmental factors or other behavioral traits are also known to affect adult body mass index (BMI) [7]. This supports the assertion that obesity is a “heritable neurobehavioral disorder that is highly sensitive to environmental conditions” [8], including in utero exposures.

Preclinical studies conducted on the impact of maternal obesity revealed that maternal obesity leads to altered neurotransmitter signaling and altered functioning especially in the striatum (the caudate nucleus and lentiform nucleus that comprise the putamen and globus pallidus) among other adverse effects such as increased anxiety [9]. The striatum, also, has recurrently been found to be involved in hedonic eating, food-related motivation, and reward processes [10–13]. Differences have been shown in neural processing of unhealthy, i.e., high-sugar content, food between healthy weight and overweight or obese individuals [14, 15]. Obesity has been associated with structural changes in the striatum in the adolescent [16] and adult population [17–19]. Moreover, the functional and structural changes in the striatum may predict future weight gain [20–23].

Given the paramount role of maternal prenatal factors that affect in utero brain development, it is crucial to investigate the neurodevelopment of the offspring who were prenatally exposed to maternal overweight/obesity. Still, there is only limited research investigating the infant neurodevelopment of those who were exposed to prenatal maternal obesity. Two extant studies have shown that alterations in functional connectivity related to reward processing and cognitive control, akin to the alterations observed in obese adults, were also identified in 2-week-old infants whose mothers had high BMI [24, 25]. In addition, decreased white matter integrity [26] and increased mean diffusivity in the hypothalamus [27, 28] were observed in infants born to mothers with high BMI.

Diffusion tensor imaging (DTI) has been an indispensable method to study the white matter and subcortical structures, since it provides quantitative scalar values to assess the microstructural properties of both white matter and subcortical structures in the brain [29–31]. The mean diffusivity (MD), the measure of free molecular motion of water in the tissue, is a scalar derived from DTI images and is regarded as the most suitable scalar for assessing gray matter structures [32]. It represents the tissue density and reflects the overall ratio of water volume between intra/extracellular tissue compartments [33]. The free-water fraction in the tissue is positively associated with the MD value and negatively associated with the presence of more cellular structures. Therefore, decreased MD has been interpreted as a marker of high tissue density [34].

Based on the structural and functional alterations observed in the striatum in obese adolescent and adult populations, the adverse effect of high maternal pre-pregnancy BMI on infant brain metrics, and the weight status of the offspring, in this study, we hypothesized that maternal pre-pregnancy BMI is positively associated with the infant striatal MD.

## Methods

### Study data

The data were collected as a part of the FinnBrain Birth Cohort Study whose broad goal is to study the effects of genes and environment on the developmental and mental health of children [35].

The study included the data of 180 infants who were recruited between 2 and 5 weeks of age (counted from the estimated due date), i.e., 1–6 weeks after birth, between 2012 and 2016. The inclusion criterion of the scanning sessions for infants was being born after gestational week 35 from singleton pregnancies. The exclusion criteria were the occurrence of any perinatal complications with potential neurological consequences and being previously diagnosed with a central nervous system anomaly or an abnormal finding in a previous magnetic resonance imaging (MRI) scan with clinical indications [36]. After the neuroimaging data preprocessing and related quality control, successful structural and diffusion MRI data of 122 infants were available for further analyses.

**Fig. 1:**
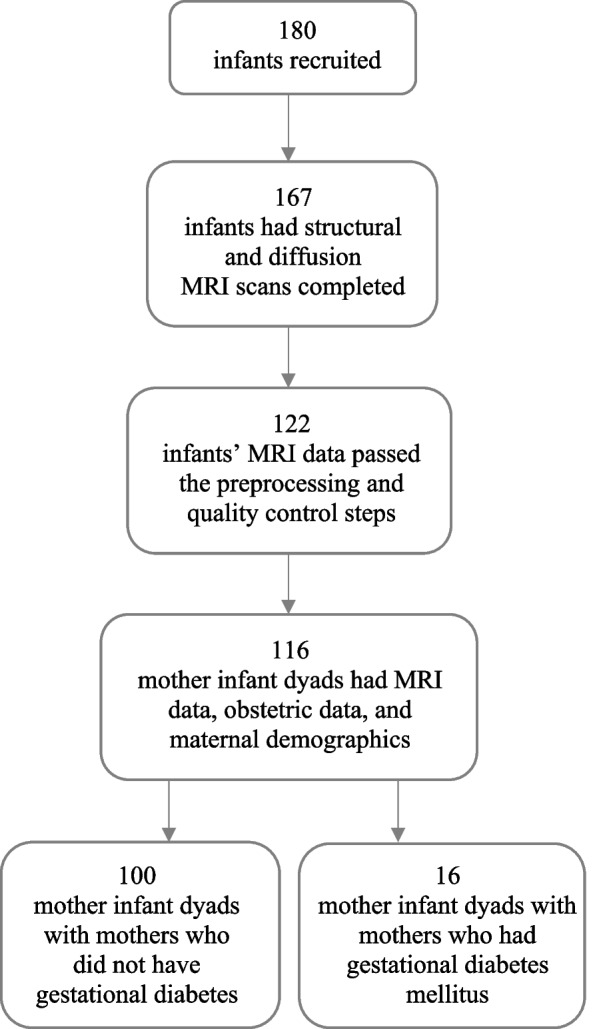
Flowchart of study sample

Obstetric data and maternal demographics were retrieved from the Finnish Medical Birth Register of the National Institute for Health and Welfare (http://www.thl.fi). The mothers had no history of alcohol or drug abuse, severe psychiatric disorders, epilepsy, or related medication use during pregnancy. Overall, the data of 116 mother-infant dyads were used in the statistical analyses (see Fig. 1).

### Image acquisition

Imaging was performed during natural sleep [37]. MRI data were acquired using a Siemens Magnetom Verio 3-T scanner (Siemens Medical Solutions, Erlangen, Germany) with a 12-element Head Matrix coil. The anatomical data were acquired using a dual-echo turbo spin echo (PD-T2-TSE) sequence (TR 12,070 ms; TE 13 ms and 102 ms) and a sagittal 3D-T1 magnetization-prepared rapid acquisition gradient echo (MPRAGE) sequence (TR 1900 ms; TE 3.26 ms; inversion time 900 ms) with whole brain coverage. A 1 × 1 × 1 mm^3^ isotropic resolution was used for both sequences.

Single-shell diffusion-weighted data was acquired using a standard twice-refocused spin echo-echo planar imaging (SE-EPI) sequence (FOV 208 mm; 64 slices; TR 9300 ms; TE 87 ms). 2 × 2 × 2 mm^3^ isotropic resolution was used for the sequence, and the *b*-value was 1000 s/mm. There were in total 96 unique diffusion encoding directions in a 3-part multi-scan DTI sequence. Each part consisted of uniformly distributed 31, 32, or 33 directions and 3 b0 images (images without diffusion encoding) that were taken at the beginning, in the middle, and at the end of each scan.

### Imaging analysis

#### Striatal segmentation

**Fig. 2:**
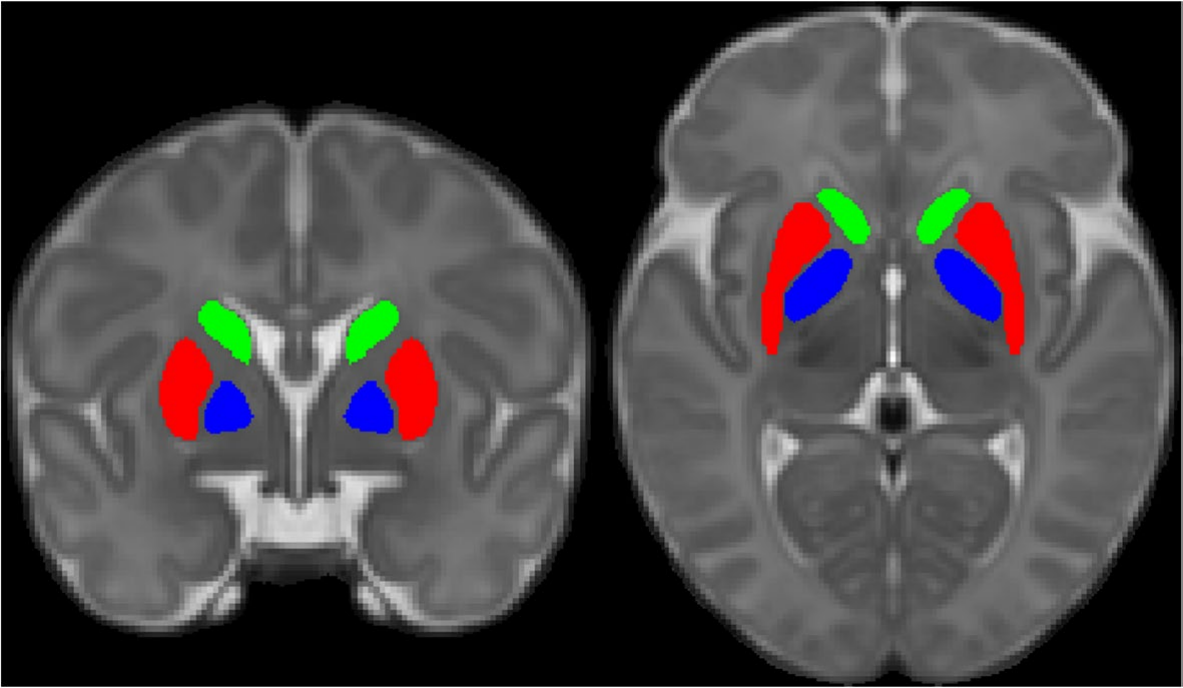
The bilateral regions of interest used in the study; caudate nucleus (in green), putamen (in red), and globus pallidus (in blue) are presented on a T2-weighted sample average brain image

A study-specific template was created from the structural MRI data of T1-weighted and T2-weighted brain images. First, a template was constructed as previously described [38]. Each subject’s T2-weighted image was coregistered to their respective T1-weighted image, and the T1-weighted image was co-registered to the MNI-152 template space to create an initial approximate template. The template was then created in iterations by minimizing the mean squared intensity difference between the template and each subject’s MRI and the magnitude of all deformation to map the template to each individual subject. Then, the data was clustered (*k* = 21) using the Jacobian determinants from the non-linear transformations derived from the template construction procedure. The central-most subject of each of the 21 clusters was chosen, and the study-specific template was warped to the central-most subject. The regions of interest (ROIs) were manually segmented in these 21 warped versions of the template. ROIs were then manually labeled on both variants of the template to ensure accuracy. Finally, the segmentations were unwarped back to the standard template and final ROI labels were created [38, 39] (see Fig. 2).

#### DTI preprocessing

Good quality b0 images were chosen manually, coregistered, averaged, and moved in front of each 4D series. Brain masks were created based on the b0 volumes with the Brain Extraction Tool (BET) [40] by FSL (FMRIB Software Library v 5.0.9 [41]. The DTIPrep software [42] was used to inspect the quality of the data. Low-quality diffusion images were discarded. The remaining images were also visually inspected, and more directions were excluded as needed. We have previously found that after the quality control steps, datasets that have more than 20 diffusion encoding directions yield reliable tensor estimates [43]. Here, all infants with at least 20 diffusion encoding directions were selected, and we used all available data thereafter. Eddy current and motion correction steps were conducted with FSL [44], and the *b*-vector matrix was rotated accordingly. A diffusion tensor model was fitted to each voxel included in the brain mask with the DTIFIT tool in FDT (FMRIB’s Diffusion Toolbox) of FSL. The DTI preprocessing steps have been provided in detail in our previous publication that also reports good test-retest repeatability in between segments of DTI sequences [45].

#### Extracting diffusion metrics

To extract the MD values of the ROIs, the diffusion data were registered to the study-specific template space. This was carried out by rigidly registering the b0 images to nonuniformity-corrected T1-weighted data and combining the transformations from b0-to-T1 and the T1-to-template space for MD maps [38, 39]. The masking of the individual structures was then performed in the group average space to get the diffusion measures. We first defined the values from the anatomical masks in the template space. Second, to eliminate the partial volume effect, 1.5-mm erosion was applied to the template masks [46]. The mean MD values within each ROI were calculated by taking the average of MD values (($\lambda 1+\lambda 2+\lambda 3$)/3) gathered as the output of the DTIFIT tool with both the non-eroded masks and the eroded masks. Here, the dataset created with values from the eroded, i.e., partial volume corrected, masks was used for the derived brain measures. The same analyses were repeated using the dataset created with values from the non-eroded masks, and the results are presented in the additional file (Additional file 1: Fig. S1, Table S1, and Table S2). In addition, BMI-stratified group difference (BMI < 25 [underweight and normal weight group] and BMI ≥ 25 [overweight and obesity group]) analysis is reported in Additional file 1: Fig. S2.

### Statistical analysis

The associations between maternal pre-pregnancy BMI and the mean MD of the ROIs were investigated with a linear regression model that was adjusted for the infant’s sex and postnatal age in days. The possible confounding effect of maternal gestational diabetes mellitus (GDM) was considered [47, 48]. The same regression model was conducted in a subsample that included only the mothers without GDM.

We performed sensitivity analyses that included other variables as covariates: infant’s birthweight and gestational age at birth in weeks, mother’s age at birth in years, socioeconomic status represented with education level (years of formal education), and the use of selective serotonin reuptake inhibitor, serotonin and norepinephrine reuptake inhibitor, or other drugs that affect the central nervous system by individually adding these factors to and removing them from the regression model, to verify that the associations were not explained by these variables. Also, sex-specific associations between maternal pre-pregnancy BMI and striatal MD were investigated.

To assure the appropriateness of the regression models, we checked the variance inflation factor (< 1.5 for all variables in all models) and visually inspected the distribution of residuals and the Q-Q plots for normality.

Multiple comparisons were corrected with the false discovery rate (FDR) correction. *P*-values less than 0.05 after FDR correction over 18 (6 ROIs × 3 variables) evaluations for multiple comparisons were considered statistically significant. All statistical analyses and data visualization were done in R 4.0.3 [49].

## Results

### Demographic overview

Demographics of infants and mothers are presented in Table 1, and the mean MD values of each of the six regions of interest are presented in Table 2. Female infants had smaller mean MD values in the right putamen; however, there were no statistically significant sex differences in the mean MD values of infants in any ROI after correcting for multiple comparisons (Table 2).

**Table 1:** Demographics of infants and mothers presented with mean scores (range) or frequencies for the whole sample (*N* = 116, 66 males 50 females)

Variable	Whole sample, *n* = 116
Birth weight (g)	3482 (2530–4700)
Birth height (cm)	50.43 (44–56)
Head circumference (cm)	35.27 (33–37.5)
Gestational weeks at birth	39.88 (36.3–42.1)
Gestational weeks at scan	43.56 (41.1–46.4)
Infant age at scan from birth (days)	25 (8 − 45)
Maternal pre-pregnancy body mass index (BMI)	24.2 (17.5 − 38.4)
Underweight (BMI < 18)	3/116
Normal weight (18 ≤ BMI < 25)	76/116
Overweight (25 ≤ BMI < 30)	25/116
Obese (BMI ≥ 30)	12/116
Gestational diabetes mellitus (insulin treatment)	16 (1)/116
SSRI/SNRI or other central nervous system-affecting drug	8/116
Maternal age at birth (years)	29.7 (20–41)
Maternal education level at birth	
< 12 years	32/116
12–15 years	36/116
15 + years	48/116

**Table 2:** Mean MD (SD) × 10^−3^ mm^2^/s values of the six regions of interest (ROIs) for the whole sample

ROI	Whole sample, *n* = 116
Right putamen	1.033 (0.030)
Left putamen	1.033 (0.030)
Right globus pallidus	1.016 (0.030)
Left globus pallidus	1.011 (0.028)
Right caudate nucleus	1.101 (0.037)
Left caudate nucleus	1.112 (0.044)

Infant age had strong negative associations with the mean MD in all ROIs after FDR correction: the right putamen (*p* for infant’s age = 2.14e − 10, partial *R*^2^ = 0.3), left putamen (*p* for infant’s age = 5.59e − 11, partial *R*^2^ = 0.32), right globus pallidus (*p* for infant’s age = 0.000248, partial *R*^2^ = 0.11), left globus pallidus (*p* for infant’s age = 0.000529, partial *R*^2^ = 0.1), right caudate nucleus (*p* for infant’s age = 0.000524, partial *R*^2^ = 0.1), and left caudate nucleus (*p* for infant’s age = 3.5e − 05, partial *R*^2^ = 0.14).

### Associations between maternal pre-pregnancy BMI and the mean MD of the striatum

**Fig. 3:**
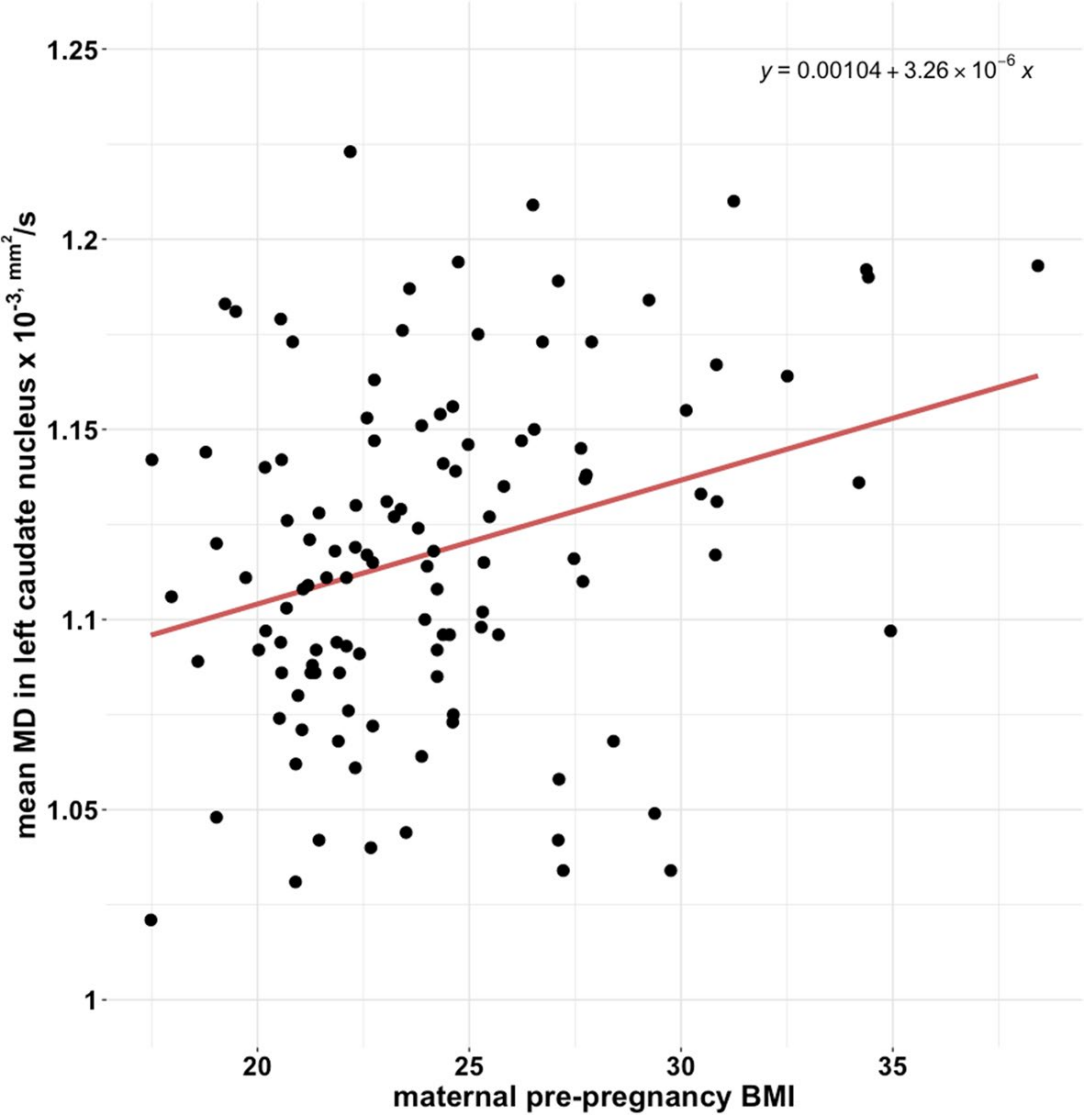
A scatterplot showing the association between maternal pre-pregnancy BMI and the mean MD in the left caudate nucleus

We found a positive association between maternal pre-pregnancy BMI and the mean MD in the left caudate nucleus (see Fig. 3 and Table 3), adjusted for the infant’s sex and postnatal age in days. Maternal pre-pregnancy BMI had no statistically significant association with the mean MD in the lentiform nuclei or the right caudate nucleus. The results remained unchanged in the sensitivity analyses. Also, there were no sex-specific associations between maternal pre-pregnancy BMI and striatal MD.

**Table 3:** The association between maternal pre-pregnancy BMI and mean MD of striatum adjusted for infant’s sex and age for the whole sample and the subsample without the gestational diabetes mellitus (GDM)

ROI	All mother-infant dyads (*n* = 116)			Mother-infant dyads without GDM (*n* = 100)		
	**Maternal pre-pregnancy BMI**			**Maternal pre-pregnancy BMI**		
	***β***	***p***	***R***^**2**^_**partial**_	***β***	***p***	**R**^**2**^_**partial**_
Right putamen	6.12 (× 10^−7^)	0.29	0.01	6.63 (× 10^−7^)	0.31	0.01
Left putamen	3.07 (× 10^−7^)	0.59	0.003	2.62 (× 10^−7^)	0.6	0.003
Right globus pallidus	1.08 (× 10^−6^)	0.1	0.02	9.53 (× 10^−7^)	0.18	0.02
Left globus pallidus	1.72 (× 10^−7^)	0.78	0.001	− 9.59 (× 10^−8^)	0.89	0.001
Right caudate nucleus	5.8 (× 10^−7^)	0.49	0.004	3.93 (× 10^−7^)	0.7	0.004
Left caudate nucleus	2.59 (× 10^−6^)	**0.006**	0.07	3.04 (× 10^−6^)	**0.004**	0.07

To assess the associations without the possible confounding effect of GDM, the cases with GDM were eliminated from the dataset (remaining *n* = 100), and the same linear regression model was applied to the subset. The results remained the same; maternal pre-pregnancy BMI was still significantly correlated with the mean MD in the left caudate nucleus but not with other striatal brain regions.

## Discussion

This study applied an ROI analysis of DTI images to investigate the association between maternal pre-pregnancy BMI and newborn offspring striatal microstructural maturation. We found that maternal pre-pregnancy BMI was positively associated with left caudate nucleus MD. The results may seem contradictory with some of the previous literature showing a negative association between maternal pre-pregnancy BMI and MD values of the offspring [50]. However, that association was found between maternal pre-pregnancy BMI and MD values of 10-year-old children and 26-year-old young adults where environmental factors such as lifestyle or socio-economic status are potentially more influential on MD values. Our study provides a conceptual advance for the investigation of in utero exposure to maternal adiposity before the abovementioned factors start to contribute.

Reward sensitivity is a critical contributor to weight gain [51] as a behavioral modifier in promoting excess caloric intake. Increased reward sensitivity to food stimuli has been linked to excessive weight gain [21]. Indeed, increased caudate nucleus activation to high sugar content food was repeatedly observed in high-risk adolescents due to parental obesity compared to low-risk adolescents [52, 53]. It can thus be speculated that the involvement of the caudate nucleus might be partly defined from the in utero exposure to maternal obesity. By showing an association between maternal BMI and infant left caudate nucleus MD, the current study give supports this hypothesis. Future longitudinal studies are critical to assess whether there is a programming effect.

The association between maternal pre-pregnancy BMI and neonate striatal MD was asymmetrical. Although it is important to note that the lack of bilateral findings may simply due to the high amount of noise present in newborn MRI [54]. However, in a deep learning study where the researchers used the anatomical scans of almost 18,000 adult participants to create a convolutional neural network to predict the BMI with high accuracy, it was found that not the right but the left caudate nucleus had a strong influence on predicting the BMI [55]. This could imply that true hemispheric asymmetry is indeed present in the studied mechanisms. Moreover, an fMRI study showed that adolescents at high risk for obesity (due to paternal obesity) showed larger right caudate nucleus responses to sugary food cues compared to the low-risk group [56]. Structural asymmetry and functional lateralization of the potential impact of parental BMI should be studied more to gain a better understanding of these results. The same applies to the possible differences between in utero exposure to maternal obesity and exposure later in life, to gain a better understanding of the neural mechanisms involved.

As opposed to the previous findings showing lowered MD values in putamen and globus pallidus in mildly obese adults [34], no such association was found with maternal adiposity and striatal MD values in neonates. However, those findings could be of postnatal environmental influence or as speculated in the related paper the association could be explained by the number of dopamine receptors in the striatal area. Takeuchi et al. argued that excessive hedonic eating is related to the increased number of dopamine receptors in the striatal areas, and the striatal axonal density is observed as lowered MD [34]. It is important to highlight the importance of future DTI and positron emission tomography multimodal imaging studies to investigate the underlying neural mechanisms of in utero exposure to obesity.

Here, we present an adverse effect of maternal pre-pregnancy BMI on the striatal development that may represent a key mechanism of offspring weight gain from birth to adulthood. Because it is well-established that primary prevention of obesity is more effective than treating adult obesity [57, 58], the investigation of alterations driven by maternal pre-pregnancy BMI in the offspring’s reward system is of high relevance to improving offspring obesity-related outcomes by planning effective interventive measures [59].

Furthermore, research shows that the adverse effect of maternal obesity on neurodevelopment manifests on a broader spectrum than problems in energy balance, including various cognitive impairments and psychiatric disorders [60, 61]. Specifically, a large meta-analysis conducted with the data of 36 cohorts from several European countries and the USA revealed that maternal obesity poses an increased risk for attention and hyperactivity disorder, autism spectrum disorders, and cognitive delay [62]. However, the causality is still speculative [63] and may be informed through the identification of mechanistic pathways such as fetal programming of reward circuitry. Thus, more research is warranted to further consider the extent to which adverse effects of in utero exposure to maternal excess BMI have on the physiological and mental well-being of future generations.

A limitation of the current study is the small number of participants with GDM. A larger sample of mothers with GDM would have provided the possibility of investigating and perhaps comparing the impact of GDM alongside BMI. Furthermore, our whole sample was drawn from the Finnish population. Similar studies with participants from more ethnically diverse backgrounds are needed to improve the generalizability of the findings. Another limitation of the study was that the paternal BMI and gestational weight gain data were not available to us to further investigate the relationship between parental BMI and offspring striatal diffusivity. Conducting a longitudinal analysis is left for future studies.

## Conclusions

The findings of this study suggest a positive association between maternal pre-pregnancy weight status and the mean MD values of the left caudate nucleus. As increased MD is commonly interpreted as lower tissue density, it could be concluded that in utero exposure to maternal obesity might have a growth/maturation impairing impact on the tissue density of the infant’s left caudate nucleus. Further investigations are required to identify the prognostic relevance of early life caudate nucleus development and weight trajectories of the offspring who were born to obese mothers.

### Supplementary Information

**Additional file 1: Fig. S1.** A scatterplot showing the association between maternal pre-pregnancy BMI and the mean MD in left caudate nucleus. **Fig. S2.** A boxplot showing the median differences of left caudate nucleus mean diffusivity between BMI < 25 group (representing underweight and normal weight classification of BMI) and BMI ≥ 25 (representing overweight and obese classification of BMI). **Table S1.** Mean MD (SD) × 10^−3^mm^2^/s values of the six regions of interest (ROIs) for whole sample using the non-eroded data. **Table S2.** The association between maternal pre-pregnancy BMI and mean MD of striatum adjusted for infant’s sex and age for the whole sample and the subsample without the gestational diabetes mellitus (GDM).

## Data Availability

The current Finnish legislation and our Ethical Board approval do not permit the open data sharing of imaging data or derived measures. Investigators interested in getting access to the data are encouraged to contact FinnBrain’s principal investigators (https://sites.utu.fi/finnbrain/en/contact/). The analysis code can be made available upon a reasonable request to the corresponding author.

## Abbreviations

BET: Brain extraction tool
BMI: Body mass index
DTI: Diffusion tensor imaging
FDR: False discovery rate
FOV: Field of view
FSL: FMRIB software library
GDM: Gestational diabetes mellitus
MD: Mean diffusivity
MPRAGE: Magnetization prepared rapid acquisition gradient echo
MRI: Magnetic resonance imaging
PD-T2-TSE: Dual-echo turbo spin echo
ROI: Region of interest
SD: Standard deviation
SE-EPI: Spin echo-echo planar imaging
TE: Echo time
TR: Repetition time

## References

[1] Whitaker RC, Wright JA, Pepe MS, Seidel KD, Dietz WH (1997). Predicting obesity in young adulthood from childhood and parental obesity. N Engl J Med.

[2] Whitaker KL, Jarvis MJ, Beeken RJ, Boniface D, Wardle J (2010). Comparing maternal and paternal intergenerational transmission of obesity risk in a large population-based sample. Am J Clin Nutr.

[3] Loos RJF, Yeo GSH (2022). Obesogenic environment. The genetics of obesity: from discovery to biology. Nat Rev Genet.

[4] Waalen J (2014). The genetics of human obesity. Transl Res.

[5] Muñoz C, Garcia-Vargas GG, Morales RP (2017). Monogenic, polygenic and multifactorial obesity in children: genetic and environmental factors. Austin J Nutr Metab.

[6] Min J, Chiu DT, Wang Y (2013). Variation in the heritability of body mass index based on diverse twin studies: a systematic review. Etiol Pathophysiol.

[7] Silventoinen K, Konttinen H (2020). Obesity and eating behavior from the perspective of twin and genetic research. Neurosci Biobehav Rev.

[8] O’Rahilly S, Farooqi IS (2008). Human obesity: a heritable neurobehavioral disorder that is highly sensitive to environmental conditions. Diabetes.

[9] Hasebe K, Kendig MD, Morris MJ. Mechanisms underlying the cognitive and behavioural effects of maternal obesity. Nutrients 2021;**13**. 10.3390/nu13010240. 10.3390/nu13010240 PMC7829712 33467657

[10] Stice E, Burger KS, Yokum S (2013). Relative ability of fat and sugar tastes to activate reward, gustatory, and somatosensory regions. Am J Clin Nutr.

[11] Luo S, Alves J, Hardy K, Wang X, Monterosso J, Xiang AH, et al. Neural processing of food cues in pre-pubertal children. Pediatr Obes 2019;14. 10.1111/IJPO.12435. 10.1111/ijpo.12435 PMC6336530 30019454

[12] Gearhardt AN, Yokum S, Harris JL, Epstein LH, Lumeng JC (2020). Neural response to fast food commercials in adolescents predicts intake. Am J Clin Nutr.

[13] Small DM, Jones-Gotman M, Dagher A (2003). Feeding-induced dopamine release in dorsal striatum correlates with meal pleasantness ratings in healthy human volunteers. Neuroimage.

[14] Geha P, Cecchi G, Todd Constable R, Abdallah C, Small DM (2017). Reorganization of brain connectivity in obesity. Hum Brain Mapp.

[15] Stoeckel LE, Weller RE, Cook EW, Twieg DB, Knowlton RC, Cox JE (2008). Widespread reward-system activation in obese women in response to pictures of high-calorie foods. Neuroimage.

[16] Kennedy JT, Collins PF, Luciana M (2016). Higher adolescent body mass index is associated with lower regional gray and white matter volumes and lower levels of positive emotionality. Front Neurosci.

[17] Dekkers IA, Jansen PR, Lamb HJ (2019). Obesity, brain volume, and white matter microstructure at MRI: a cross-sectional UK biobank study. Radiology.

[18] Samara A, Li Z, Rutlin J, Raji CA, Sun P, Song SK (2021). Nucleus accumbens microstructure mediates the relationship between obesity and eating behavior in adults. Obesity.

[19] Kim AY, Shim JH, Choi HJ, Baek HM (2020). Comparison of volumetric and shape changes of subcortical structures based on 3-dimensional image between obesity and normal-weighted subjects using 3.0 T MRI. J Clin Neurosci.

[20] Stice E, Burger K (2019). Neural vulnerability factors for obesity. Clin Psychol Rev.

[21] Stice E, Yokum S (2021). Neural vulnerability factors that predict future weight gain. Curr Obes Rep.

[22] Nakamura Y, Ozawa S, Koike S (2020). Caudate functional connectivity associated with weight change in adolescents. Front Hum Neurosci.

[23] Yokum S, Gearhardt AN, Harris JL, Brownell KD, Stice E (2014). Individual differences in striatum activity to food commercials predict weight gain in adolescents. Obesity (Silver Spring).

[24] Salzwedel AP, Gao W, Andres A, Badger TM, Glasier CM, Ramakrishnaiah RH (2019). Maternal adiposity influences neonatal brain functional connectivity. Front Hum Neurosci.

[25] Li X, Andres A, Shankar K, Pivik RT, Glasier CM, Ramakrishnaiah RH (2016). Differences in brain functional connectivity at resting state in neonates born to healthy obese or normal-weight mothers. Int J Obes.

[26] Ou X, Thakali KM, Shankar K, Andres A, Badger TM (2015). Maternal adiposity negatively influences infant brain white matter development. Obesity.

[27] Rasmussen JM, Tuulari JJ, Nolvi S, Thompson PM, Merisaari H, Lavonius M (2023). Maternal pre-pregnancy body mass index is associated with newborn offspring hypothalamic mean diffusivity: a prospective dual-cohort study. BMC Med.

[28] Rasmussen JM, Thompson PM, Gyllenhammer LE, Lindsay KL, O’Connor TG, Koletzko B (2022). Maternal free fatty acid concentration during pregnancy is associated with newborn hypothalamic microstructure in humans. Obesity.

[29] Assaf Y, Johansen-Berg H, Thiebaut de Schotten M (2019). The role of diffusion MRI in neuroscience. NMR Biomed.

[30] Assaf Y (2019). Imaging laminar structures in the gray matter with diffusion MRI. Neuroimage.

[31] Takeuchi H, Kawashima R (2018). Mean diffusivity in the dopaminergic system and neural differences related to dopaminergic system. Curr Neuropharmacol.

[32] Hashempour N, Tuulari JJ, Merisaari H, Acosta H, Lewis JD, Pelto J (2023). Prenatal maternal depressive symptoms are associated with neonatal left amygdala microstructure in a sex-dependent way. Eur J Neurosci.

[33] Sagi Y, Tavor I, Hofstetter S, Tzur-Moryosef S, Blumenfeld-Katzir T, Assaf Y (2012). Learning in the fast lane: new insights into neuroplasticity. Neuron.

[34] Takeuchi H, Taki Y, Nouchi R, Yokoyama R, Nakagawa S, Iizuka K (2020). The associations of BMI with mean diffusivity of basal ganglia among young adults with mild obesity and without obesity. Sci Rep.

[35] Karlsson L, Tolvanen M, Scheinin NM, Uusitupa HM, Korja R, Ekholm E (2018). Cohort profile: the FinnBrain Birth Cohort Study (FinnBrain). Int J Epidemiol.

[36] Lehtola SJ, Tuulari JJ, Karlsson L, Parkkola R, Merisaari H, Saunavaara J (2019). Associations of age and sex with brain volumes and asymmetry in 2–5-week-old infants. Brain Struct Funct.

[37] Copeland A, Silver E, Korja R, Lehtola SJ, Merisaari H, Saukko E (2021). Infant and child MRI: a review of scanning procedures. Front Neurosci.

[38] Acosta H, Kantojärvi K, Hashempour N, Pelto J, Scheinin NM, Lehtola SJ (2020). Partial support for an interaction between a polygenic risk score for major depressive disorder and prenatal maternal depressive symptoms on infant right amygdalar volumes. Cereb Cortex.

[39] Lewis JD, Fonov VS, Collins DL, Evans AC, Tohka J (2019). Cortical and subcortical T1 white/gray contrast, chronological age, and cognitive performance. Neuroimage.

[40] Smith SM (2002). Fast robust automated brain extraction. Hum Brain Mapp.

[41] Jenkinson M, Beckmann CF, Behrens TEJ, Woolrich MW, Smith SM (2012). FSL. Neuroimage.

[42] Oguz I, Farzinfar M, Matsui J, Budin F, Liu Z, Gerig G (2014). DTIPrep: quality control of diffusion-weighted images. Front Neuroinform.

[43] Giannelli M, Cosottini M, Michelassi MC, Lazzarotti G, Belmonte G, Bartolozzi C (2010). Dependence of brain DTI maps of fractional anisotropy and mean diffusivity on the number of diffusion weighting directions. J Appl Clin Med Phys.

[44] Andersson JLR, Sotiropoulos SN (2016). An integrated approach to correction for off-resonance effects and subject movement in diffusion MR imaging. Neuroimage.

[45] Merisaari H, Tuulari JJ, Karlsson L, Scheinin NM, Parkkola R, Saunavaara J (2019). Test-retest reliability of diffusion tensor imaging metrics in neonates. Neuroimage.

[46] Douaud G, Behrens TE, Poupon C, Cointepas Y, Jbabdi S, Gaura V (2009). In vivo evidence for the selective subcortical degeneration in Huntington’s disease. Neuroimage.

[47] Kim SY, England JL, Sharma JA, Njoroge T, Alexander B (2011). Maternal gestational diabetes mellitus and long-term risk of childhood obesity and childhood diabetes: a systematic-review. Exp Diabetes Res.

[48] Kawasaki M, Arata N, Miyazaki C, Mori R, Kikuchi T, Ogawa Y et al. Obesity and abnormal glucose tolerance in offspring of diabetic mothers: a systematic review and meta-analysis. PLoS One 2018;13. 10.1371/journal.pone.0190676. 10.1371/journal.pone.0190676 PMC5766126 29329330

[49] RStudio Team (2020). RStudio: integrated development for R.

[50] Verdejo-Román J, Björnholm L, Muetzel RL, Torres-Espínola FJ, Lieslehto J, Jaddoe V (2018). Maternal prepregnancy body mass index and offspring white matter microstructure: results from three birth cohorts. Int J Obes.

[51] Davis C, Strachan S, Berkson M (2004). Sensitivity to reward: implications for overeating and overweight. Appetite.

[52] Shearrer GE, Stice E, Burger KS (2018). Adolescents at high risk of obesity show greater striatal response to increased sugar content in milkshakes. Am J Clin Nutr.

[53] Stice E, Yokum S, Burger KS, Epstein LH, Small DM (2011). Youth at risk for obesity show greater activation of striatal and somatosensory regions to food. J Neurosci.

[54] Dubois J, Alison M, Counsell SJ, Hertz-Pannier L, Hüppi PS, Benders MJNL. MRI of the neonatal brain: a review of methodological challenges and neuroscientific advances. 2020.10.1002/jmri.27192. 10.1002/jmri.27192 PMC8247362 32420684

[55] Vakli P, Deák-Meszlényi RJ, Auer T, Vidnyánszky Z. Predicting body mass index from structural MRI brain images using a deep convolutional neural network. Front Neuroinform 2020;14. 10.3389/FNINF.2020.00010. 10.3389/fninf.2020.00010 PMC7104804 32265681

[56] Sadler JR, Shearrer GE, Papantoni A, Yokum ST, Stice E, Burger KS (2023). Correlates of neural adaptation to food cues and taste: the role of obesity risk factors. Soc Cogn Affect Neurosci.

[57] Pandita A, Sharma D, Pandita D, Pawar S, Tariq M, Kaul A (2016). Childhood obesity: prevention is better than cure. Diabetes Metab Syndr Obes Targets Ther.

[58] Lanigan J. Prevention of overweight and obesity in early life. 10.1017/S0029665118000411. 10.1017/S0029665118000411 29808786

[59] Cirulli F, Musillo C, Berry A (2020). Maternal obesity as a risk factor for brain development and mental health in the offspring. Neuroscience.

[60] Contu L, Hawkes CA. A review of the impact of maternal obesity on the cognitive function and mental health of the offspring. Int J Mol Sci 2017;18. 10.3390/ijms18051093. 10.3390/ijms18051093 PMC5455002 28534818

[61] Elshenawy S, Simmons R (2016). Maternal obesity and prenatal programming. Mol Cell Endocrinol.

[62] Sanchez CE, Barry C, Sabhlok A, Russell K, Majors A, Kollins SH (2018). Maternal pre-pregnancy obesity and child neurodevelopmental outcomes: a meta-analysis. Obes Rev.

[63] Torres-Espínola FJ, Berglund SK, García-Valdés LM, Segura MT, Jerez A, Campos D (2015). Maternal obesity, overweight and gestational diabetes affect the offspring neurodevelopment at 6 and 18 months of age - a follow up from the PREOBE cohort. PLoS ONE.

