# Supplementary material for: Associations between maternal pre-pregnancy BMI and infant striatal mean diffusivity
Source: BMC Med. 2024 Mar 25;22:140. doi: 10.1186/s12916-024-03340-z (PMC10964641; doi:10.1186/s12916-024-03340-z)
Supplement: Supplementary file 1 — Additional file 1: Fig. S1. A scatterplot showing the association between maternal pre-pregnancy BMI and the mean MD in left caudate nucleus. Fig. S2. A boxplot showing the median differences of left caudate nucleus mean diffusivity between BMI < 25 group (representing underweight and normal weight classification of BMI) and BMI ≥ 25 (representing overweight and obese classification of BMI). Table S1. Mean MD (SD) × 10−3mm2/s values of the six regions of interest (ROIs) for whole sample using the non-eroded data. Table S2. The association between maternal pre-pregnancy BMI and mean MD of striatum adjusted for infant’s sex and age for the whole sample and the subsample without the gestational diabetes mellitus (GDM). [file 12916_2024_3340_MOESM1_ESM.docx]

**Associations between maternal pre-pregnancy BMI and infant striatal mean diffusivity**

**Authors:** Aylin Rosberg^a,b^, Harri Merisaari^a,e^, John D. Lewis^f^, Niloofar Hashempour^a^, Minna Lukkarinen^a,c^, Jerod M. Rasmussen^g^, Noora M. Scheinin^a,b^, Linnea Karlsson^a,c^, Hasse Karlsson^a,b,h^, Jetro J. Tuulari^a,b,d^

**We report two additional results:**

**1. The regression results that were obtained without performing a partial volume correction to the anatomical labels as stated in the main manuscript: “**The masking of the individual structures was then performed in the group average space to get the diffusion measures. We first defined the values from the anatomical masks in the template space. Second, to eliminate the partial volume effect, 1.5 mm erosion to the template masks. Mean MD values within each ROI was calculated with non-eroded masks and eroded masks. Here, the dataset created with values from the eroded, *i.e.* partial volume corrected, masks was used for the derived brain measures. The same analyses were repeated using the dataset created with values from the non-eroded masks and the results are presented in the additional file (Additional file 1: Figure S1, Table S1 and Table S2). In addition, BMI stratified group difference (BMI < 25 [underweight and normal weight group] and BMI ≥ 25 [overweight and obesity group]) analysis is reported in the additional file (Additional File 1: Figure S2).**”**

**The overall pattern of results is identical although differences are also apparent.**

**Table S1**. Mean MD (SD) × 10^-3^ mm^2^/s values of the six regions of interest (ROIs) for whole sample using the non-eroded data.

| **ROI** | **whole sample**  n = 116 |
| --- | --- |
| right  putamen | 1.044 (0.029) |
| left  putamen | 1.046 (0.029) |
| right globus pallidus | 1.018 (0.028) |
| left globus pallidus | 1.014 (0.026) |
| right  caudate nucleus | 1.161 (0.046) |
| left  caudate nucleus | 1.119 (0.063) |

A significant positive association was found between maternal pre-pregnancy BMI and the mean MD in the left caudate nucleus (see Figure S1 and Table S2). The results remained unchanged in the sensitivity analyses.

**Figure S1.** ﻿A scatterplot showing the association between maternal pre-pregnancy BMI and the mean MD in left caudate nucleus.

**Table S2**. The association between maternal pre-pregnancy BMI and mean MD of striatum adjusted for infant’s sex and age for the whole sample and the subsample without the gestational diabetes mellitus (GDM).

|  | **all mother-infant**  **dyads (n = 116)** | | | | | **mother-infant dyads**  **without GDM**  **(n = 100)** | | |
| --- | --- | --- | --- | --- | --- | --- | --- | --- |
|  | | **Maternal pre-pregnancy BMI** | | | **Maternal pre-pregnancy BMI** | | | |
| **ROI** | | ﻿ 𝜷 | ***p*** | **R^2^ _partial_** | 𝜷 | | ***p*** | **R^2^ _partial_** |
| right  putamen | | 4.3(x10^-7^) | 0.75 | 0.005 | 4.07(x10^-7^) | | 0.53 | 0.004 |
| left  putamen | | 2.07(x10^-7^) | 0.72 | 0.001 | 2.51(x10^-7^) | | 0.7 | 0.002 |
| right globus pallidus | | 9.53(x10^-6^) | 0.13 | 0.02 | 7.9(x10^-7^) | | 0.24 | 0.01 |
| left globus pallidus | | 1.18(x10^-7^) | 0.84 | 0.0004 | -1.42(x10^-7^) | | 0.83 | 0.001 |
| right  caudate nucleus | | 1.65(x10^-6^) | 0.33 | 0.01 | 9.83(x10^-7^) | | 0.44 | 0.001 |
| left  caudate nucleus | | 4.3(x10^-6^) | **0.002** | 0.08 | 5.29(x10^-6^) | | **0.0008** | 0.11 |

**2. Maternal pre-pregnancy BMI stratified analysis**

The Wilcoxon rank sum test showed a significant difference in medians of left caudate nucleus mean MD values between group BMI < 25 (n = 79, median = 0.001109), representing underweight and normal weight classification of BMI, and BMI ≥ 25 (n = 37, median = 0.001136), representing overweight and obese classification of BMI, *p* = 0.009; r = .24 (see Figure S2).

**Figure S2.** A boxplot showing the median differences of left caudate nucleus mean diffusivity between BMI < 25 group (representing underweight and normal weight classification of BMI) and BMI ≥ 25 (representing overweight and obese classification of BMI).
